# Generation of bipartite entanglement in a dissipative cavity magnomechanical system

**DOI:** 10.1038/s41598-025-12942-3

**Published:** 2025-07-24

**Authors:** Hamid Reza Baghshahi, Mohammad Javad Faghihi, Mahboobeh Moslehi

**Affiliations:** 1 Department of Physics, Faculty of Science, Vali-e-Asr University of Rafsanjan, Rafsanjan, Iran; 2 Department of Photonics, Graduate University of Advanced Technology, Kerman, Iran

**Keywords:** Entanglement, Magnonics, Optomechanics, Kerr nonlinearity, Dissipation, Quantum optics, Quantum information

## Abstract

In this work, we employ logarithmic negativity to rigorously investigate bipartite entanglements in a lossy cavity magnomechanical system incorporating both photon and magnon Kerr nonlinearities. The system comprises two optical cavity modes, two yttrium-iron-garnet (YIG) spheres, which support magnon and phonon modes, and two electromagnetic fields that drive the magnons. Through numerical simulations, we systematically examine the influence of significant parameters, including photon-magnon and phonon-magnon coupling strengths, dissipation rates, Kerr nonlinearities, environmental temperatures, and normalized detuning on the bipartite entanglements between distinct subsystems. Our findings reveal that the amounts of bipartite entanglements can be precisely tuned by optimizing these parameters. Specifically, increasing either dissipation or Kerr nonlinearity diminishes the maximum values of entanglement. Furthermore, when the magnomechanical coupling is stronger, the entanglement becomes more robust and can endure across a broader spectrum of temperatures. Moreover, the entanglement generated within the subsystems demonstrates remarkable robustness against environmental temperature. Additionally, the maximum survival temperature of bipartite entanglements varies across different entangled pairs, and can be effectively controlled by the optical-magnon coupling strength. Notably, entanglement between subsystems persists even at cryogenic temperatures.

## Introduction

Entanglement is one of the most fascinating aspects of quantum physics, serving as a foundational resource for quantum information processing, communication, and sensing applications^1,2^. This phenomenon occurs when two or more systems become connected such that the state of one instantly influences the state of the other, no matter how far apart they are^3^. Understanding how entanglement is created in different systems is therefore critical for advancing quantum technologies. In cavity quantum electrodynamics, one of the major focuses is the study of hybrid quantum systems, where magnons, photons, and mechanical vibrations interact to generate rich quantum effects^4–8^.

Over the past decade, hybrid quantum systems, particularly those involving magnons, that is, collective spin excitations in magnetic materials like yttrium-iron-garnet (YIG, $\mathrm {Y_{3}Fe_{5}O_{12}}$), have received significant attention owing to their high spin density ($\approx 4.22 \times 10^{27}$ m^−3^) and low damping rate ($\approx 1 \textrm{MHz}$)^9–11^. These properties make magnons ideal system to couple to cavities and superconducting qubits^12,13^. Moreover, in these systems that magnons interact with both optical and mechanical degrees of freedom, strong quantum correlations can be created, leading to the observation of some quantum phenomena such as optical cooling of magnon^14^ magnon-polariton bistability^15^, and magnomechanically induced transparency^16^. Additionally, hybrid magnonic systems can, for instance, be utilized for ultrasensitive magnetometry^17^, quantum state storage^18^, and magnon-assisted photon-phonon conversion^19^.

Magnon-based systems are becoming highly significant in quantum technology because they can create and control quantum entanglement. For instance, in a system that combines optical, electronic, and magnonic components, light and microwave signals can become entangled^20^. Another exciting approach involves cavity magnonics, where strong quantum entanglement can be generated through a special magnonical converter^21^. Additionally, it has been reported that magnonic and mechanical systems can work together. By linking them with optical pulses, large-scale entanglement can be generated, enabling the transfer of quantum information between magnonic and long-lived mechanical nodes^22^. Recently, it has been shown that how magnons behave in a specific type of ferromagnet when exposed to heat^23^.

One of the issue in this field is that how to create large-scale quantum entanglement using cavity optomagnonics. In this regard, a study has explored three-way entanglement in cavity systems^24^ and successfully created high-fidelity Bell states, indicating the strongest magnon-photon entanglement^25^. Also, recent research highlights advancements in quantum entanglement, specifically demonstrating its generation and control in hybrid cavity-magnon-phonon systems via photon tunneling^26^, and the enhancement of magnon-magnon entanglement using an optical parametric amplifier in a double-cavity setup^27^

In particular, and in direct relation to the present work, it has been shown that quantum entanglement can be enhanced by utilizing Kerr nonlinearity, an effect caused by magnetocrystalline anisotropy. This effect improves steady-state entanglement between two or three quantum systems^28^ and has even been applied to generate magnon-magnon entangled states^29^. Additionally, some studies have explored how magnons and photons become entangled in cavity magnonic systems through magnonic Kerr nonlinearity^30^.

Another important consideration is the influence of the surrounding environment, particularly in magnonic systems. For instance, the dynamics of entanglement between magnons in certain cavity systems^31^ and photon-magnon entanglement have been investigated in energy-dissipating environments^32^. Notably, experiments have also been conducted to track how entanglement behaves in cavity magnonics, especially when magnons and photons interact in ways that lead to energy loss^33^. Furthermore, different types of interactions in dissipative cavity magnonics have been studied and compared to better understand their respective advantages and disadvantages^34^.

Motivated by these considerations, we examine entanglement across six subsystem pairs (photon–photon, photon–magnon, photon–phonon, magnon–magnon, magnon–phonon, phonon–phonon) in a dissipative cavity magnomechanical system featuring both photonic and magnonic Kerr nonlinearities. The system consists of two optical cavity modes, two YIG spheres, each supporting magnon and phonon modes, and two driving electromagnetic fields that excite the magnon modes, with inherent dissipation from optical, magnonic, and phononic channels. To quantify bipartite entanglement, we employ logarithmic negativity, an entanglement monotone valid under both local operations with classical communication (LOCC) and positive partial transpose (PPT) preserving operations^35,36^. It should be noted that this measure also represents an upper bound for distillable entanglement and relates to entanglement cost under PPT operations^37,38^. Through numerical analysis, we investigate how key parameters including photon–magnon and phonon–magnon coupling strengths, dissipation rates, Kerr nonlinearities, environmental temperature, and the normalized detuning influence entanglement between subsystems.

The remainder of this paper is organized as follows. Section 2 presents a detailed description of the system. Section 3 derives the equations of motion governing the system dynamics. We then analyze the degree of bipartite entanglements between subsystems in Sect. 4. In Sect. 5, we present and discuss numerical results examining how system parameters affect both the strength and range of entanglement. Finally, Sect. 6 provides concluding remarks and summarizes our key findings.

## Theoretical model

**Fig. 1:**
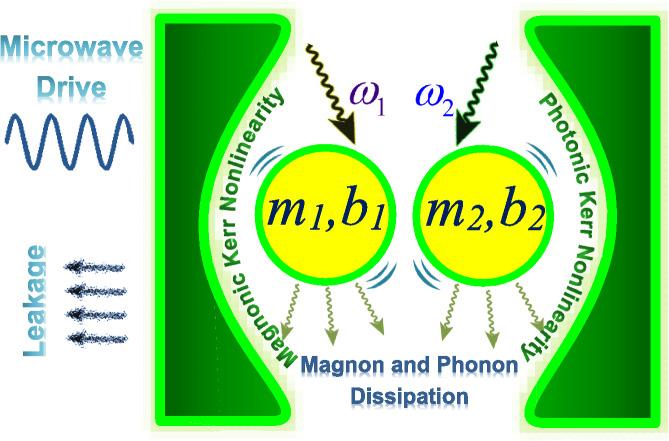
The schematic depicts a cavity magnomechanical system where two YIG spheres interact with the optical cavity modes. The microcavity is formed by a high-reflectivity mirror and a partially transmitting mirror, the latter enabling photon leakage from the system.

We consider a cavity magnomechanical system composed of two microwave cavity modes, two magnon modes, and two vibrational phonon modes, featuring both optical and magnonic Kerr nonlinearities, as illustrated in Fig. 1. The magnon modes are driven by two electromagnetic fields, coupling to microwave cavity photons via magnetic-dipole interaction and to phonon modes in the YIG spheres via magnetostrictive interaction. The total system Hamiltonian is given by ($\hbar = 1$)

1 $$\begin{aligned} \hat{H}= &\, \sum _{j=1}^{2}\Bigg (\omega _{a_{j}}\hat{a}_{j}^{\dagger }\hat{a}_{j}+\omega _{m_{j}} \hat{m}_{j}^{\dagger }\hat{m}_{j}+\frac{1}{2}\omega _{b_{j}}(\hat{q}_{j}^{2}+\hat{p}_{j}^{2})+g_{m_{j}b_{j}} \hat{m}_{j}^{\dagger }\hat{m}_{j}\hat{q}_{j}\Bigg ) \\ &+ \sum _{j=1}^2\sum _{k=1}^2g_{jk} \left( \hat{a}_{k}\hat{m}_{j}^\dagger +\hat{a}_{k}^\dagger \hat{m}_{j} \right) + \sum _{j=1}^{2}\chi _{m_{j}}\hat{m}_{j}^{\dagger }\hat{m}_{j}\hat{m}_{j}^{\dagger } \hat{m}_{j} + \sum _{j=1}^{2}\chi _{a_{j}}\hat{a}_{j}^\dagger {}^{2}\hat{a}_{j}^{2}+i\sum _{j=1}^{2}\Omega _{j} \left( \hat{m}_{j}^{\dagger } e^{-i\omega _{d_{j}}t}-\hat{m}_{j}e^{i\omega _{d_{j}}t} \right) , \end{aligned}$$

where $\hat{a}_{j}$ ($\hat{a}_{j}^{\dagger }$) and $\hat{m}_{j}$ ($\hat{m}_{j}^{\dagger }$) ($j=1,2$) are the annihilation (creation) operators for the photon and magnon modes, respectively, while $\hat{q}_{j}$ and $\hat{p}_{j}$ represent the dimensionless position and momentum quadratures of the mechanical modes. The resonance frequencies of the optical, magnon, and mechanical modes are given by $\omega _{a_{j}}$, $\omega _{m_{j}}$, and $\omega _{b_{j}}$, respectively. The coupling strengths $g_{jk}$ and $g_{m_{j}b_{j}}$ describe the photon-magnon and phonon-magnon interactions, respectively. The parameter $\Omega _{j}$ corresponds to the Rabi frequency between the magnon modes and external driving fields with frequency $\omega _{d_{j}}$, while $\chi _{m_{j}}$ and $\chi _{a_{j}}$ denote the self-Kerr nonlinear coefficients for the magnon and cavity modes, respectively. The Hamiltonian comprises several distinct contributions: terms 1–3 correspond to the free Hamiltonians of the optical, magnon, and phonon modes; terms 4–5 describe the magnon-phonon and magnon-optical interactions; terms 6–7 represent the magnonic and optical Kerr nonlinearities; and term 8 accounts for the driving field-magnon mode interaction.

In the frame rotating at the magnon drive frequency $\omega _{d_{j}}$, the Hamiltonian of the system under the condition $\omega _{d_{1}} = \omega _{d_{2}}$ can be obtained as

2 $$\begin{aligned} \hat{\mathscr {H}}= &\, \sum _{j=1}^{2}\Bigg (\Delta _{a_{j}}\hat{a}_{j}^{\dagger }\hat{a}_{j}+\Delta _{m_{j}} \hat{m}_{j}^{\dagger }\hat{m}_{j}+\frac{1}{2}\omega _{b_{j}}(\hat{q_{j}}^{2}+\hat{p_{j}}^{2})+g_{m_{j}b_{j}} \hat{m}_{j}^{\dagger }\hat{m}_{j}\hat{q_{j}}\Bigg ) \\& + \sum _{j=1}^2\sum _{k=1}^2g_{jk} \left( \hat{a}_{k}\hat{m}_{j}^\dagger +\hat{a}_{k}^\dagger \hat{m}_{j} \right) + \sum _{j=1}^{2}\chi _{m_{j}}\hat{m}_{j}^{\dagger }\hat{m}_{j}\hat{m}_{j}^{\dagger } \hat{m}_{j} + \sum _{j=1}^{2}\chi _{a_{j}}\hat{a}_{j}^\dagger {}^{2}\hat{a}_{j}^{2}+i\sum _{j=1}^{2}\Omega _{j} \left( \hat{m}_{j}^{\dagger }-\hat{m}_{j} \right) , \end{aligned}$$

where $$\Delta _{a_{j}(m_{j})}=\omega _{a_{j}(m_{j})}-\omega _{d_{j}}$$.

## Equations of motion

Employing the Hamiltonian from Eq. 2 and accounting for various sources of dissipation, we derive the system dynamics through the following set of quantum Langevin equations:

3a $$\begin{aligned} \dot{\hat{a}}_{j}= &\, -(i\Delta _{a_{j}}+\gamma _{a_{j}}) \hat{a}_{j}-2i\chi _{a_{j}} \hat{a}_{j}^\dagger {} \hat{a}_{j}^{2} - i\sum _{k=1}^{2}g_{kj} \hat{m}_{k}+\sqrt{2\gamma _{a_{j}}} \hat{a}_{j}^{\textrm{in}}, \end{aligned}$$

3b $$\begin{aligned} \dot{\hat{m}}_{j}= &\, -(i\Delta _{m_{j}}+\gamma _{m_{j}}+ig_{m_{j}b_{j}}q_{j}) \hat{m}_{j}-2i\chi _{m_{j}} \hat{m}_{j}^\dagger {} \hat{m}_{j} \hat{m}_{j} - i\sum _{k=1}^{2}g_{jk} \hat{a}_{k}+\Omega _{j}+\sqrt{2\gamma _{m_{j}}} \hat{m}_{j}^{\textrm{in}}, \end{aligned}$$

3c $$\begin{aligned} \dot{\hat{q}}_{j}= &\, \omega _{b_{j}}\hat{p}_{j}, \end{aligned}$$

3d $$\begin{aligned} \dot{\hat{p}}_{j}= &\, -\omega _{b_{j}}\hat{q}_{j}-g_{m_{j}b_{j}}\hat{m}_{j}^\dagger {} \hat{m}_{j}-\gamma _{b_{j}}\hat{p}_{j}+\hat{\xi }_{j}, \end{aligned}$$

where $\gamma _{a_{j}}$, $\gamma _{m_{j}}$, and $\gamma _{b_{j}}$ represent the dissipation rates of the $j\textrm{th}$ photon, magnon, and phonon modes, respectively. The input noise operators $\hat{a}_{j}^{\textrm{in}}$, $\hat{m}_{j}^{\textrm{in}}$, and $\hat{\xi }_{j}$ correspond to the photon, magnon, and phonon modes, respectively. These noise operators have zero mean values and obey the correlation relations as follows

4a $$\begin{aligned} \langle \hat{\mathscr {O}}^{\textrm{in}}{}^\dagger {}(t) \hat{\mathscr {O}}^{\textrm{in}}(t')\rangle= &\, N_{\mathscr {O}}(\omega _{\mathscr {O}})\delta (t-t'), \end{aligned}$$

4b $$\begin{aligned} \langle \hat{\mathscr {O}}^{\textrm{in}}(t) \hat{\mathscr {O}}^{\textrm{in}}{}^\dagger {}(t')\rangle= &\, \left( N_{\mathscr {O}}(\omega _{\mathscr {O}}) + 1 \right) \delta (t-t'), \nonumber \\ ~(\hat{\mathscr {O}}=\hat{a}_{j},\hat{m}_{j}) \end{aligned}$$

and

5 $$\begin{aligned} \langle \hat{\xi }_{j}(t) \hat{\xi }_{j}(t')+ \hat{\xi }_{j}(t') \hat{\xi }_{j}(t)\rangle /2=\, & \gamma _{b_{j}}(2N_{b_{j}}(\omega _{b_{j}})+1) \nonumber \\& \times \delta (t-t'), \end{aligned}$$

where $N_{\mathscr {O}}(\omega _{\mathscr {O}}) = \left[ \exp \left( \hbar \omega _{\mathscr {O}}/k_{B}T\right) - 1\right] ^{-1}$ (for $\mathscr {O} = a_{j}, m_{j}, b_{j}$) represents the mean thermal occupation number for the cavity, magnon, and phonon modes at environmental temperature *T*, with $k_{B}$ denoting the Boltzmann constant.

Since the magnon modes are strongly driven, they acquire large steady-state amplitudes $|\langle \hat{m}_{j} \rangle | \gg 1$. Through the linear cavity-magnon coupling, the optical modes consequently develop similarly large amplitudes $|\langle \hat{a}_{j} \rangle | \gg 1$. This allows us to linearize the system dynamics about the steady-state solutions by decomposing each operator as $\hat{\mathscr {O}}(t) = \langle \hat{\mathscr {O}} \rangle + \delta \hat{\mathscr {O}}(t), (\hat{\mathscr {O}} = \hat{m}_{j}, \hat{a}_{j}, \hat{q}_{j}, \hat{p}_{j}),$ while safely neglecting higher-order fluctuation terms. The quantum Langevin equations governing the fluctuation dynamics are then derived from Eq. 3 as follows

6a $$\begin{aligned} \hspace{-0.5cm}\delta \dot{\hat{a}}_{j}= &\, -(i\Delta _{a_{j}}+\gamma _{a_{j}})\delta \hat{a}_{j}-i\tilde{\chi }_{a_{j}}(\delta \hat{a}_{j}^{\dagger }+2\delta \hat{a}_{j}) - i\sum _{k=1}^{2}g_{kj}\delta \hat{m}_{k}+\sqrt{2\gamma _{a_{j}}}\delta \hat{a}_{j}^{\textrm{in}}, \end{aligned}$$

6b $$\begin{aligned} \hspace{-0.5cm}\delta \dot{\hat{m}}_{j}= &\, -(i\tilde{\Delta }_{m_{j}}+\gamma _{m_{j}})\delta \hat{m}_{j}-i\tilde{\chi }_{m_{j}}(\delta \hat{m}_{j}^{\dagger }+2\delta \hat{m}_{j}) - i\sum _{k=1}^{2}g_{jk}\delta \hat{a}_{k}-\frac{1}{\sqrt{2}}G_{m_{j}b_{j}}\delta \hat{q}_{j}+\sqrt{2\gamma _{m_{j}}}\delta \hat{m}_{j}^{\textrm{in}}, \end{aligned}$$

6c $$\begin{aligned} \hspace{-0.5cm}\delta \dot{\hat{q}}_{j}= &\, \omega _{b_{j}}\delta \hat{p}_{j}, \end{aligned}$$

6d $$\begin{aligned} \hspace{-0.5cm}\delta \dot{\hat{p}}_{j}= &\, -\omega _{b_{j}}\delta q_{j}+G_{m_{j}b_{j}}\delta \hat{Y}_{m_{j}}-\gamma _{b_{j}}\delta \hat{p}_{j}, \end{aligned}$$

where $\tilde{\chi }_{\mathscr {O}} = 2\chi _{\mathscr {O}}|\langle \hat{\mathscr {O}} \rangle |^2$ (for $\hat{\mathscr {O}}=\hat{a}_j,\hat{m}_j$) is the effective self-Kerr coefficient, $\tilde{\Delta }_{m_j} = \Delta _{m_j} + g_{m_jb_j}\langle \hat{q}_j \rangle$ is the effective magnon-drive detuning, and $G_{m_jb_j} = i\sqrt{2}g_{m_jb_j}\langle \hat{m}_j \rangle$ represents the effective magnomechanical coupling. The steady-state expectation values of the system operators are given by

7a $$\begin{aligned} \langle \hat{m}_{j}\rangle= &\, \frac{\Omega _{j}-i\displaystyle {\sum _{k=1}^{2}g_{jk}\langle \hat{a}_{k}\rangle }}{i (\tilde{\Delta }_{m_{j}}+\tilde{\chi }_{m_{j}})+\gamma _{m_{j}}}, \end{aligned}$$

7b $$\begin{aligned} \langle \hat{a}_{j}\rangle= &\, \frac{-i\displaystyle {\sum _{k=1}^{2}g_{kj}\langle \hat{m}_{k}\rangle }}{i(\Delta _{a_{j}}+\tilde{\chi }_{a_{j}})+\gamma _{a_{j}}}, \end{aligned}$$

7c $$\begin{aligned} \langle \hat{q}_{j}\rangle= &\, \frac{-g_{m_{j}b_{j}}|\langle \hat{m}_{j}\rangle |^{2}}{\omega _{b_{j}}}, \end{aligned}$$

7d $$\begin{aligned} \langle \hat{p}_{j}\rangle= &\, 0. \end{aligned}$$

In deriving these relations, we note that since $\langle \hat{a}_j \rangle$ and $\langle \hat{m}_j \rangle$ are approximately real-valued, we have the approximations $\tilde{\chi }_{a_j} \simeq 2\chi _{a_j}|\langle \hat{a}_j \rangle |^2$ and $\tilde{\chi }_{m_j} \simeq 2\chi _{m_j}|\langle \hat{m}_j \rangle |^2$.

## Bipartite entanglements

In this section, we study the bipartite entanglements between different subsystems by means of the logarithmic negativity measure^39^. This measure, suggested by Vidal and Werner, can be assessed through the quantum fluctuations of the system’s quadratures. A set of quadrature fluctuation and noise operators can be defined as^40^

8a $$\begin{aligned} \delta \hat{X}_{\mathscr {O}}&= \frac{\delta \hat{\mathscr {O}}^{\dagger }+\delta \hat{\mathscr {O}}}{\sqrt{2}}, \quad \quad ~ \delta \hat{Y}_{\mathscr {O}} = i\frac{\delta \hat{\mathscr {O}}^{\dagger }-\delta \hat{\mathscr {O}}}{\sqrt{2}}, \end{aligned}$$

8b $$\begin{aligned} \delta \hat{X}_{\mathscr {O}}^{\textrm{in}}&= \frac{\delta \hat{\mathscr {O}}^{\dagger \textrm{in}}+\delta \hat{\mathscr {O}}^{\textrm{in}}}{\sqrt{2}}, \quad \delta \hat{Y}_{\mathscr {O}}^{\textrm{in}} = i\frac{\delta \hat{\mathscr {O}}^{\dagger \textrm{in}}-\delta \hat{\mathscr {O}}^{\textrm{in}}}{\sqrt{2}}, \end{aligned}$$

where $\hat{\mathscr {O}} = \hat{a}_{j}, \hat{m}_{j}, \hat{q}_{j}, \hat{p}_{j}$. The linearized quantum Langevin equations for the above quadrature fluctuation operators can be written in compact form as

9 $$\begin{aligned} \dot{\hat{u}}(t) = M \hat{u}(t) + \hat{\Lambda }(t), \end{aligned}$$

where $\hat{u}(t)$ and $\hat{\Lambda }(t)$ are the vectors for quantum fluctuation and noise, respectively, defined as

10 $$\begin{aligned} \hat{u}(t) = \begin{bmatrix} \delta \hat{X}_{a_{1}}, \delta \hat{Y}_{a_{1}}, \delta \hat{X}_{a_{2}}, \delta \hat{Y}_{a_{2}}, \\ \delta \hat{X}_{m_{1}}, \delta \hat{Y}_{m_{1}}, \delta \hat{X}_{m_{2}}, \delta \hat{Y}_{m_{2}}, \\ \delta \hat{q}_{1}, \delta \hat{p}_{1}, \delta \hat{q}_{2}, \delta \hat{p}_{2} \end{bmatrix}^{\intercal }_{1 \times 12}, \end{aligned}$$

and

11 $$\begin{aligned} \hat{\Lambda }(t) = \begin{bmatrix} \sqrt{2\gamma _{a_{1}}} \hat{X}_{a_{1}}^{\textrm{in}}, \sqrt{2\gamma _{a_{1}}} \hat{Y}_{a_{1}}^{\textrm{in}}, \\ \sqrt{2\gamma _{a_{2}}} \hat{X}_{a_{2}}^{\textrm{in}}, \sqrt{2\gamma _{a_{2}}} \hat{Y}_{a_{2}}^{\textrm{in}}, \\ \sqrt{2\gamma _{m_{1}}} \hat{X}_{m_{1}}^{\textrm{in}}, \sqrt{2\gamma _{m_{1}}} \hat{Y}_{m_{1}}^{\textrm{in}}, \\ \sqrt{2\gamma _{m_{2}}} \hat{X}_{m_{2}}^{\textrm{in}}, \sqrt{2\gamma _{m_{2}}} \hat{Y}_{m_{2}}^{\textrm{in}}, 0, \hat{\xi }_{1}, 0, \hat{\xi }_{2} \end{bmatrix}^{\intercal }_{1 \times 12}. \end{aligned}$$

The drift matrix $M$ is given by

12 $$\begin{aligned} M &= \left( \begin{array}{cccccc} -\gamma _{a_{1}}& \Delta _{a_{1}}+\tilde{\chi }_{a_{1}}& 0& 0& 0& g_{11}\\-(\Delta _{a_{1}}+3\tilde{\chi }_{a_{1}})& -\gamma _{a_{1}}& 0& 0& -g_{11}& 0\\ 0& 0& -\gamma _{2}& \Delta _{a_{2}}+\tilde{\chi }_{a_{2}}& 0& g_{12}\\0& 0& -(\Delta _{a_{2}}+3\tilde{\chi }_{a_{2}})& -\gamma _{a_{2}}& -g_{12}& 0\\ 0& g_{11}& 0& g_{12}& -\gamma _{m_{1}}& \tilde{\Delta }_{m_{1}}+\tilde{\chi }_{m_{1}}\\ -g_{11}& 0& -g_{12}& 0& -(\tilde{\Delta }_{m_{1}}+3\tilde{\chi }_{m_{1}})& -\gamma _{m_{1}}\\ 0& g_{21}& 0& g_{22}& 0& 0\\ -g_{12}& 0& -g_{22}& 0& 0& 0\\ 0& 0& 0& 0& 0& 0\\ 0& 0& 0& 0& 0& G_{m_{1}b_{1}}\\ 0& 0& 0& 0& 0& 0\\ 0& 0& 0& 0& 0& 0\\\end{array} \right. \\ & \quad \left. \begin{array}{cccccc} 0& g_{21}& 0& 0& 0& 0 \\ -g_{21}& 0& 0& 0& 0& 0\\ 0& g_{22}& 0& 0& 0& 0\\-g_{22}& 0& 0& 0& 0& 0\\ 0& 0& -G_{m_{1}b_{1}}& 0& 0& 0\\ 0& 0& 0& 0& 0& 0\\ -\gamma _{m_{2}}& \tilde{\Delta }_{m_{2}}+\tilde{\chi }_{m_{2}}& 0& 0& -G_{m_{2}b_{2}}& 0\\ -(\tilde{\Delta }_{m_{2}}+3\tilde{\chi }_{m_{2}})& -\gamma _{m_{2}}& 0& 0& 0& 0\\ 0& 0& 0& \omega _{b_{1}}& 0& 0\\0& 0& -\omega _{b_{1}}& -\gamma _{b_{1}}& 0& 0\\ 0& 0& 0& 0& 0& \omega _{b_{2}}\\ 0& G_{m_{2}b_{2}}& 0& 0& -\omega _{b_{2}}& -\gamma _{b_{2}} \\\end{array} \right) . \end{aligned}$$

Due to the linearized dynamics and the Gaussian nature of all noise sources, the system’s dynamical map preserves the Gaussian character of any input state. As a result, the steady state of quantum fluctuations corresponds to a continuous-variable four-mode Gaussian state, completely characterized by a $12 \times 12$ covariance matrix *V* with elements defined as

13 $$\begin{aligned} V_{ij}(t,t') = \frac{1}{2} \langle \hat{\sigma }_i(t) \hat{\sigma }_j(t') + \hat{\sigma }_j(t') \hat{\sigma }_i(t) \rangle . \end{aligned}$$

The steady-state covariance matrix *V* obeys the Lyapunov equation as follows^40^

14 $$\begin{aligned} MV + VM^\textsf{T} = -D, \end{aligned}$$

where *D* represents the diffusion matrix given by

15 $$\begin{aligned} D = \text {diag} \begin{bmatrix} \gamma _{a_{1}} (2N_{a_{1}}+1), \gamma _{a_{1}} (2N_{a_{1}}+1), \\ \gamma _{a_{2}} (2N_{a_{2}}+1), \gamma _{a_{2}} (2N_{a_{2}}+1), \\ \gamma _{m_{1}} (2N_{m_{1}}+1), \gamma _{m_{1}} (2N_{m_{1}}+1), \\ \gamma _{m_{2}} (2N_{m_{2}}+1), \gamma _{m_{2}} (2N_{m_{2}}+1), \\ 0, \gamma _{b_{1}}(2N_{b_{1}}+1), \\ 0, \gamma _{b_{2}}(2N_{b_{2}}+1) \end{bmatrix}_{1 \times 12}. \end{aligned}$$

**Fig. 2:**
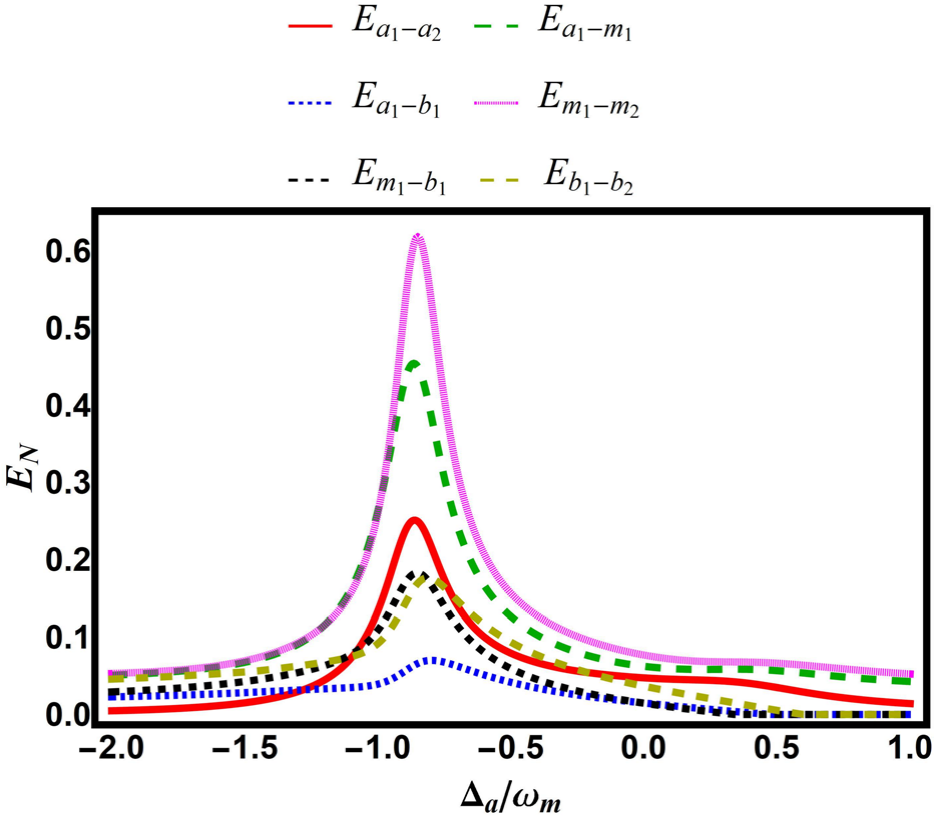
Bipartite entanglement versus normalized detuning $\Delta _a/\omega _b$, with parameters provided in Table 1.

To quantify bipartite entanglement, we employ the logarithmic negativity $E_N$^39^, defined as

16 $$\begin{aligned} E_N = \max \big [0, -\ln (2\eta ^-)\big ], \end{aligned}$$

in which the eigenvalue $\eta ^-$ is given by

17 $$\begin{aligned} \eta ^- \equiv \sqrt{\sum V - \left[ \sum V^2 - 4 \det V \right] ^{1/2}} \Big / \sqrt{2}, \end{aligned}$$

with $\det V$ and $\sum V$ being the two symplectic invariants of the reduced covariance matrix *V* of two modes, defined as^41^

18 $$\begin{aligned} \sum V \equiv \det A + \det B - 2\det C, \end{aligned}$$

where

19 $$\begin{aligned} V = \begin{bmatrix} A & C \\ C^{\intercal } & B \end{bmatrix}. \end{aligned}$$

Here, *A* and *B* are the local covariance matrices of the subsystems, while *C* encodes their cross-correlations.

## Numerical results

**Fig. 3:**
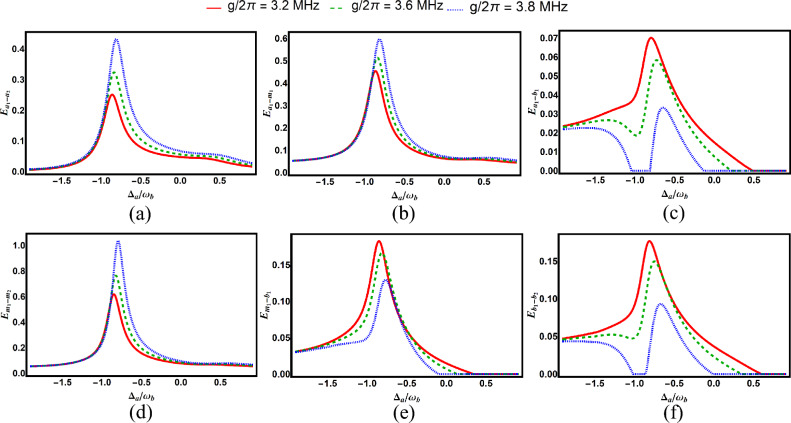
Degree of entanglement versus normalized detuning $\Delta _a/\omega _b$ for various subsystem pairs under different photon-magnon coupling strengths: (**a**) cavity–cavity ($E_{a_1-a_2}$), (**b**) cavity–magnon ($E_{a_1-m_1}$), (**c**) cavity–phonon ($E_{a_1-b_1}$), (**d**) magnon–phonon ($E_{m_1-b_1}$), (**e**) magnon–magnon ($E_{m_1-m_2}$), and (**f**) phonon–phonon ($E_{b_1-b_2}$). Other parameters are identical to those in Fig. 2.

**Fig. 4:**
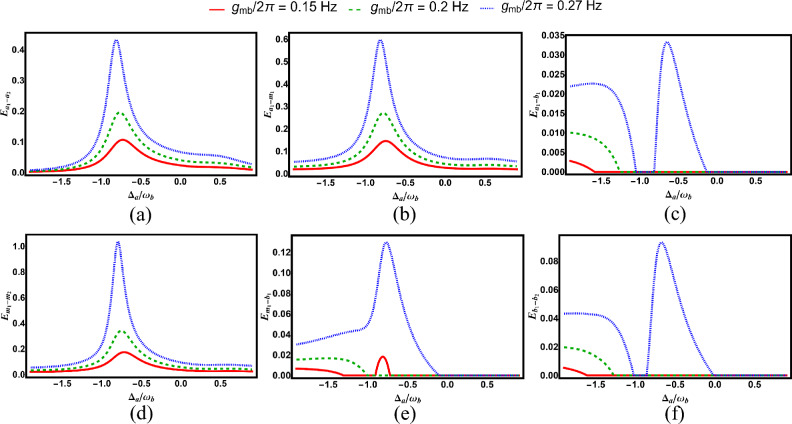
Degree of entanglement versus normalized detuning $\Delta _a/\omega _b$ for various subsystem pairs under different magnomechanical coupling strengths: (**a**) cavity–cavity ($E_{a_1-a_2}$), (**b**) cavity–magnon ($E_{a_1-m_1}$), (**c**) cavity–phonon ($E_{a_1-b_1}$), (**d**) magnon–phonon ($E_{m_1-b_1}$), (**e**) magnon–magnon ($E_{m_1-m_2}$), and (**f**) phonon–phonon ($E_{b_1-b_2}$). Other parameters are identical to those in Fig. 2.

**Fig. 5:**
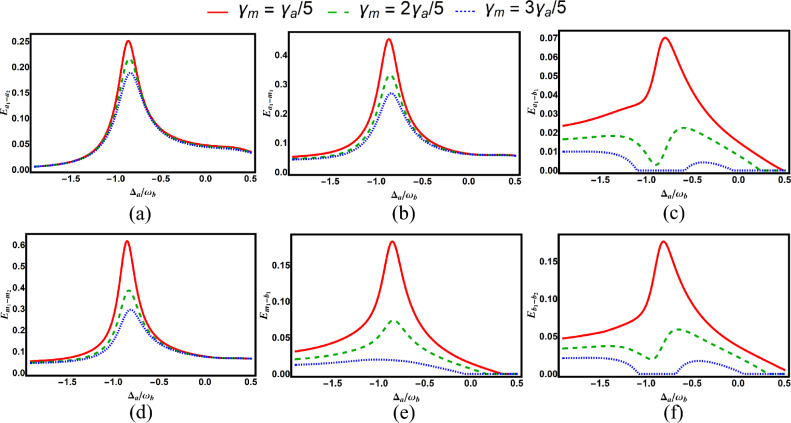
Degree of entanglement versus normalized detuning $\Delta _a/\omega _b$ for various subsystem pairs under different values of dissipation parameter of magnon: (**a**) cavity–cavity ($E_{a_1-a_2}$), (**b**) cavity–magnon ($E_{a_1-m_1}$), (**c**) cavity–phonon ($E_{a_1-b_1}$), (**d**) magnon–phonon ($E_{m_1-b_1}$), (**e**) magnon–magnon ($E_{m_1-m_2}$), and (**f**) phonon–phonon ($E_{b_1-b_2}$). Other parameters are identical to those in Fig. 2.

**Fig. 6:**
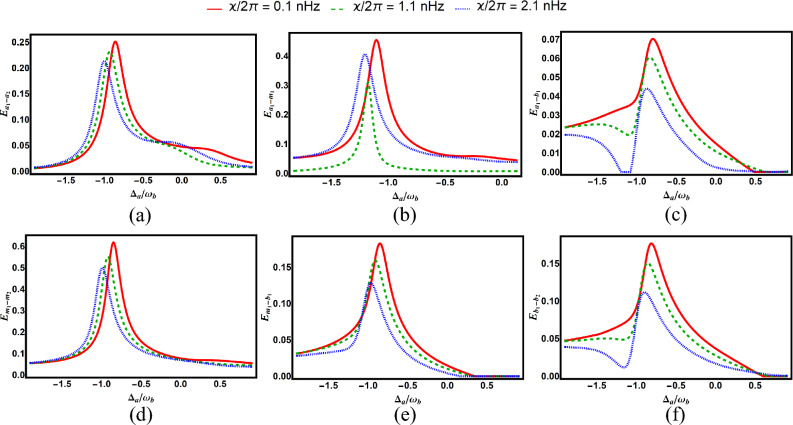
Degree of entanglement versus normalized detuning $\Delta _a/\omega _b$ for various subsystem pairs under different values of Kerr nonlinearity: (**a**) cavity–cavity ($E_{a_1-a_2}$), (**b**) cavity–magnon ($E_{a_1-m_1}$), (**c**) cavity–phonon ($E_{a_1-b_1}$), (**d**) magnon–phonon ($E_{m_1-b_1}$), (**e**) magnon–magnon ($E_{m_1-m_2}$), and (**f**) phonon–phonon ($E_{b_1-b_2}$). Other parameters are identical to those in Fig. 2.

**Fig. 7:**
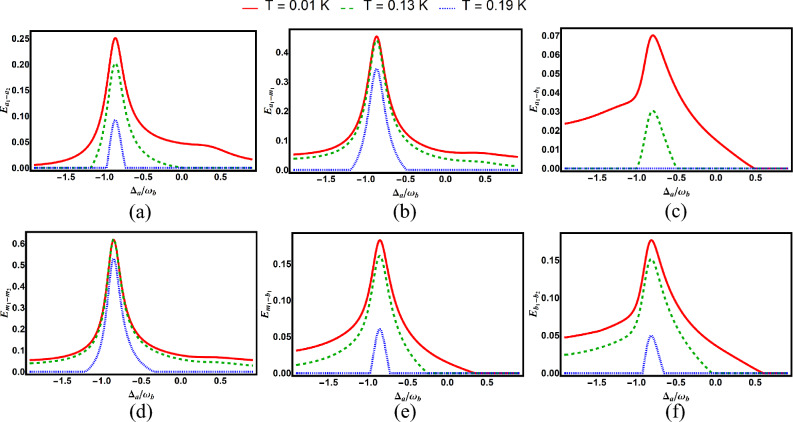
Degree of entanglement versus normalized detuning $\Delta _a/\omega _b$ for various subsystem pairs under different values of environmental temperature with $\Delta _{a}=-0.86\omega _{b}$: (**a**) cavity–cavity ($E_{a_1-a_2}$), (**b**) cavity–magnon ($E_{a_1-m_1}$), (**c**) cavity–phonon ($E_{a_1-b_1}$), (**d**) magnon–phonon ($E_{m_1-b_1}$), (**e**) magnon–magnon ($E_{m_1-m_2}$), and (**f**) phonon–phonon ($E_{b_1-b_2}$). Other parameters are identical to those in Fig. 2.

**Fig. 8:**
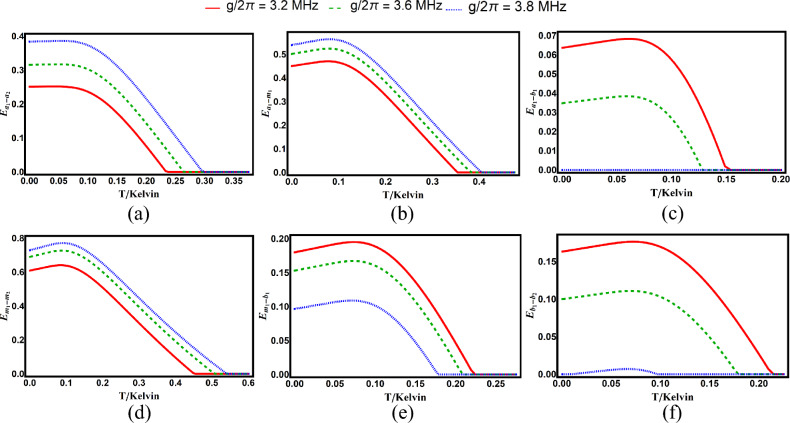
Degree of entanglement versus environmental temperature with $\Delta _{a}=-0.86\omega _{b}$ for various subsystem pairs under different values of photon–magnon coupling strength: (**a**) cavity–cavity ($E_{a_1-a_2}$), (**b**) cavity–magnon ($E_{a_1-m_1}$), (**c**) cavity–phonon ($E_{a_1-b_1}$), (**d**) magnon–phonon ($E_{m_1-b_1}$), (**e**) magnon–magnon ($E_{m_1-m_2}$), and (**f**) phonon–phonon ($E_{b_1-b_2}$). Other parameters are identical to those in Fig. 2.

**Fig. 9:**
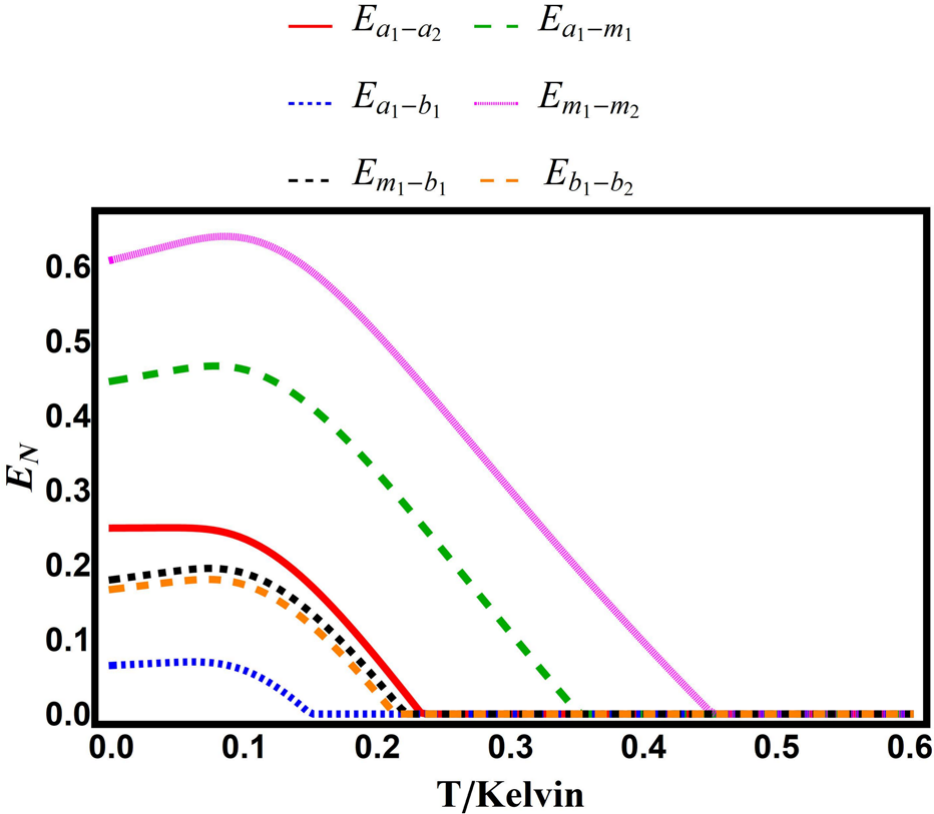
Bipartite entanglements versus environmental temperature with $\Delta _{a}=-0.86\omega _{b}$. Other parameters are identical to those in Fig. 2.

The dynamics of bipartite entanglements are presented in Figs. 2, 3, 4, 5, 6, 7, 8, 9 for various parameter regimes. To simplify the analysis while maintaining the generality of the model, we consider symmetric parameters for all subsystems. Furthermore, we adopt experimentally realistic parameter values in our numerical simulations, consistent with previous experimental work^24^. These parameters are summarized in Table 1.

**Table 1:** Description of experimentally feasible parameters used in numerical calculations, as justified by Ref. ^24^.

Parameter	Value
Symmetric parameters (for $j=1,2$)	
Photon–magnon coupling	$g_{jk} = g$
Magnonic Kerr coefficient	$\tilde{\chi }_{m_j} = \tilde{\chi }_m$
Optical Kerr coefficient	$\tilde{\chi }_{a_j} = \tilde{\chi }_a$
Magnonic dissipation rate	$\gamma _{m_j} = \gamma _m$
Optical dissipation rate	$\gamma _{a_j} = \gamma _a$
Phononic dissipation rate	$\gamma _{b_j} = \gamma _b$
Magnon detuning	$\tilde{\Delta }_{m_j} = \tilde{\Delta }_m$
Cavity detuning	$\Delta _{a_j} = \tilde{\Delta }_a$
Optical-mode frequency	$\omega _{a_j} = \omega _a$
Magnon-mode frequency	$\omega _{m_j} = \omega _m$
Phonon-mode frequency	$\omega _{b_j} = \omega _b$
Magnomechanical coupling	$g_{m_jb_j} = g_{mb}$
Numerical values	
Cavity/magnon frequency	$\omega _a/2\pi = \omega _m/2\pi = 10$ GHz
Phonon frequency	$\omega _b/2\pi = 10$ MHz
Phonon dissipation	$\gamma _b/2\pi = 100$ Hz
Cavity dissipation	$\gamma _a/2\pi = 3$ MHz
Magnon dissipation	$\gamma _m = \gamma _a/5$
Photon-magnon coupling	$g/2\pi = 3.2$ MHz
Magnomechanical coupling	$g_{mb}/2\pi = 0.27$ Hz
Temperature	$T = 10$ mK
Kerr coefficients	$\tilde{\chi }_a/2\pi = \tilde{\chi }_m/2\pi = 0.1$ nHz
Magnon detuning	$\tilde{\Delta }_m = 0.9\omega _b$

Figure 2 shows the variation of bipartite entanglements as a function of normalized detuning $\Delta _a/\omega _b$. The results demonstrate that entanglement between subsystems can be controlled by tuning the detuning parameter. Specifically, the maximum entanglement occurs between magnon modes ($E_{m_1-m_2}$), while the minimum appears between photon and phonon modes ($E_{a_1-b_1}$). The figure also reveals that increasing the detuning reduces entanglement strength, particularly for photon-phonon, magnon-phonon, and phonon-phonon pairs.

Figure 3 examines the effect of photon–magnon coupling strength on bipartite entanglements. We find that increasing the coupling enhances both the depth and the domain of $E_{a_1a_2}$, $E_{a_1m_1}$, and $E_{m_1m_2}$, while reducing these characteristics for $E_{a_1b_1}$, $E_{m_1b_1}$, and $E_{b_1b_2}$.

Figure 4 illustrates the influence of magnomechanical coupling strength on bipartite entanglement, for a fixed value of $g/2\pi = 3.8\,\text {MHz}$, using the same parameters as in Fig. 2. As shown, increasing the magnomechanical coupling enhances both the strength (depth) and the temperature range (domain) over which bipartite entanglement persists.

Figure 5 shows the role of the magnon dissipation parameter in bipartite entanglements for fixed *g* and parameters from Fig. 2. Increasing the magnon decay rate $\gamma _{m}$ reduces both the magnitude and range of entanglement, particularly for magnon-phonon and phonon-phonon entanglements.

Figure 6, which concentrates on the effect optical and magnon Kerr nonlinearities, displays the temporal evolution of bipartite entanglements versus scaled time $\Delta _{a}/\omega _{b}$. The maximum entanglement decreases with increasing Kerr nonlinearity parameters.

Figure 7 demonstrates the effect of environment temperature on bipartite entanglements. Higher temperatures reduce entanglement strength, especially for photon-magnon pairs, consistent with thermal decoherence effects in quantum systems.

The temperature robustness of bipartite entanglements for different photon-magnon couplings *g* is shown in Fig. 8. Increasing *g* enhances (suppresses) entanglement and survival temperature for photon-photon/photon-magnon/magnon-magnon (photon-phonon/magnon-phonon/phonon-phonon) entanglements. For fixed *g*, the maximum (minimum) survival temperature corresponds to magnon-magnon (photon-phonon) entanglement.

Figure 9 examines the case $g/2\pi =3.2\,\textrm{MHz}$. Magnon-magnon entanglement survives up to $0.45\,\textrm{K}$, while photon-phonon entanglement persists only to $0.15\,\textrm{K}$.

## Conclusion

In this study, we investigated quantum entanglement in a cavity magnomechanical system consisting of two microwave cavity modes, two magnon modes, and two mechanical vibrational modes with both optical and magnonic Kerr nonlinearities. By analyzing quantum fluctuations around the steady state using quantum Langevin equations, we characterized entanglement between all subsystem pairs: cavity-cavity ($E_{a_1-a_2}$), cavity-magnon ($E_{a_1-m_1}$), cavity-phonon ($E_{a_1-b_1}$), magnon-magnon ($E_{m_1-m_2}$), magnon-phonon ($E_{m_1-b_1}$), and phonon-phonon ($E_{b_1-b_2}$), quantified through logarithmic negativity. The numerical results demonstrate precise control of entanglement strength and temperature resilience through four key parameters: (1) photon-magnon coupling strength *g*, (2) phonon-magnon coupling strength $g_{mb}$, (3) magnon decay rate $\gamma _m$, (4) Kerr nonlinearities $\chi _a$ and $\chi _m$, (5) environmental temperature *T*, and (6) the normalized detuning $\Delta _a/\omega _b$. Most remarkably, magnon-magnon entanglement maintains quantum correlations up to 0.45 K, while cavity-phonon entanglement proves most sensitive to thermal noise, vanishing below 0.15 K. Moreover, as the magnomechanical coupling increases, bipartite entanglement becomes stronger and remains robust over a wider temperature range. Also, the degree of entanglement is strongly influenced by the normalized detuning $(\Delta _a/\omega _b)$. We have observed that the maximum entanglement for various entangled pairs occurs at specific detuning values, such as around $\Delta _a/\omega _b \approx -0.86$. Generally, increasing the detuning beyond these optimal points leads to a reduction in entanglement strength. These findings provide important insights for developing quantum technologies, particularly for quantum memory and signal conversion applications where magnon-based entanglement shows superior performance. The study offers practical approaches for optimizing quantum effects in experimental setups with realistic conditions.

## Data Availability

The data that support the findings of this study are available within this article.
